# The impact of the Xpert MTB/RIF screening among hospitalized patients with pneumonia on timely isolation of patients with pulmonary tuberculosis

**DOI:** 10.1038/s41598-020-79639-7

**Published:** 2021-01-18

**Authors:** Seung Beom Han, Joonhong Park, Seul Ki Ji, So Hee Jang, Soyoung Shin, Myung Sook Kim, Seung Soo Kim, Sun Hee Park

**Affiliations:** 1 Infection Prevention and Control Unit, The Catholic University of Korea, Daejeon St. Mary’s Hospital, Daejeon, Korea; 2 Department of Pediatrics, College of Medicine, The Catholic University of Korea, Seoul, Korea; 3 Department of Laboratory Medicine, College of Medicine, The Catholic University of Korea, Seoul, Korea; 4 Division of Pulmonology, Department of Internal Medicine, College of Medicine, The Catholic University of Korea, Seoul, Korea; 5 Division of Infectious Diseases, Department of Internal Medicine, College of Medicine, The Catholic University of Korea, Seoul, Korea

**Keywords:** Tuberculosis, Health policy

## Abstract

In South Korea where the tuberculosis (TB) burden is intermediate, the risk of in-hospital transmission of TB remains high. We conducted a retrospective cohort study of 244 inpatients diagnosed with pulmonary TB (2015–2018) to evaluate the impact of the Xpert MTB/RIF assay (Xpert) screening on timely isolation. TB screening was performed with smear microscopy and a polymerase chain reaction test, and the Xpert was additionally used from November 2016. Among all patients with pulmonary TB, the median time-to-isolation was significantly reduced (22.6 vs. 69.7 h; *p* < 0.001) and segmented regression analysis adjusting for the time trend showed a reduction in time-to-isolation with the introduction of the Xpert (− 39.3 h; 95% CI − 85.6, 7.0; *p* = 0.096). Among 213 patients who were timely screened (≤ 72 h after admission), time-to-isolation decreased significantly (− 38.2 h; 95% CI − 70.6, − 5.8; *p* = 0.021) with the introduction of the Xpert, and its decreasing trend continued. The Xpert provided a shorter turnaround time (4.8 vs. 49.1 h; *p* < 0.001) and higher sensitivity (76.6% vs. 47.8%; *p* < 0.001) than smear microscopy. Thus, the Xpert can be a useful screening test for pulmonary TB in real-life hospital settings with an intermediate TB burden.

## Introduction

Tuberculosis (TB) is one of the most common infectious diseases worldwide. Despite the effort to control TB, the disease burden in South Korea is intermediate with the incidence of TB being 51.5/100,000 population in 2018^1^. The number of Air-borne Infection Isolation Rooms (AIIRs) is limited in the majority of Korean hospitals, and therefore, patients with respiratory symptoms are usually hospitalized in multi-bed rooms despite continuous admission of patients with pulmonary TB. If patients are not clinically suspected to have pulmonary TB, they usually remain in multi-bed rooms until they are diagnosed with pulmonary TB, increasing the risk of TB exposure in other patients and healthcare workers (HCWs)^2^. However, predicting pulmonary TB based on patients’ symptoms, signs, and chest X-ray (CXR) findings is unsatisfactory^3^, and it is particularly challenging among old individuals as they often present with atypical symptoms and radiological findings^4^. Therefore, microbiological screening for pulmonary TB in patients with respiratory symptoms and abnormal CXR findings is critical for the timely identification and isolation of patients with pulmonary TB. Smear microscopy has been performed as a conventional screening modality for TB; however, its low sensitivity (48.9–67.1%) and possible false positivity in patients with non-tuberculous *Mycobacterium* infection are concerns^5,6^. The Xpert MTB/RIF assay (Cepheid Inc., Sunnyvale, CA, USA; Xpert), which is a rapid, automated, and cartridge-based real-time polymerase chain reaction (PCR) test, has been endorsed by the World Health Organization and is widely used for detecting *Mycobacterium tuberculosis* (MTB)^7^. Because the Xpert can deliver a result of MTB detection and rifampin resistance in approximately 2 h with higher sensitivity than conventional smear microscopy, its utility has been explored in various clinical settings with high or low TB burden^6,8,9^.

**Figure 1:**
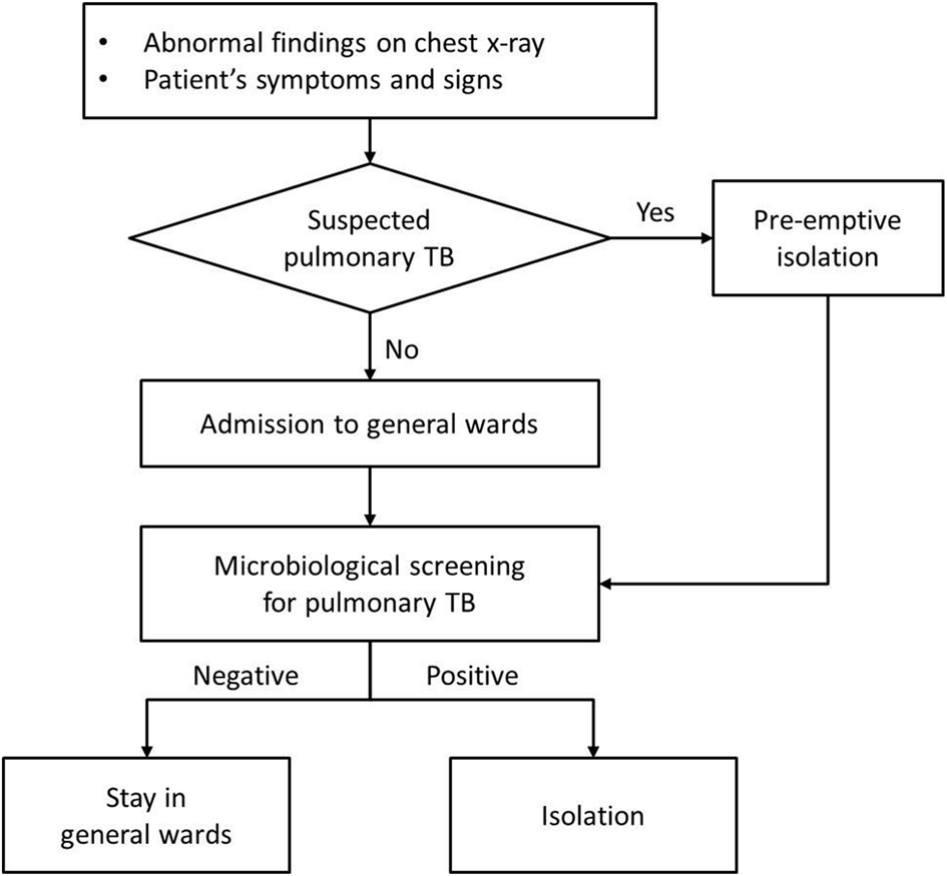
Hospital’s tuberculosis screening strategy for hospitalized patients with pneumonia.

Early identification and timely isolation of inpatients with pulmonary TB is a key to limit in-hospital TB transmission. In line with this purpose, a screening strategy among patients hospitalized with pneumonia has been implemented in our hospital since January 2015 to detect pulmonary TB regardless of clinical or radiological suspicion (Fig. 1). In addition to conventional screening tests, the Xpert was introduced in November 2016. This study aimed to evaluate the impact of the Xpert as a screening tool on early identification and isolation of patients with pulmonary TB in real-life hospital settings with an intermediate TB burden.

## Results

**Figure 2:**
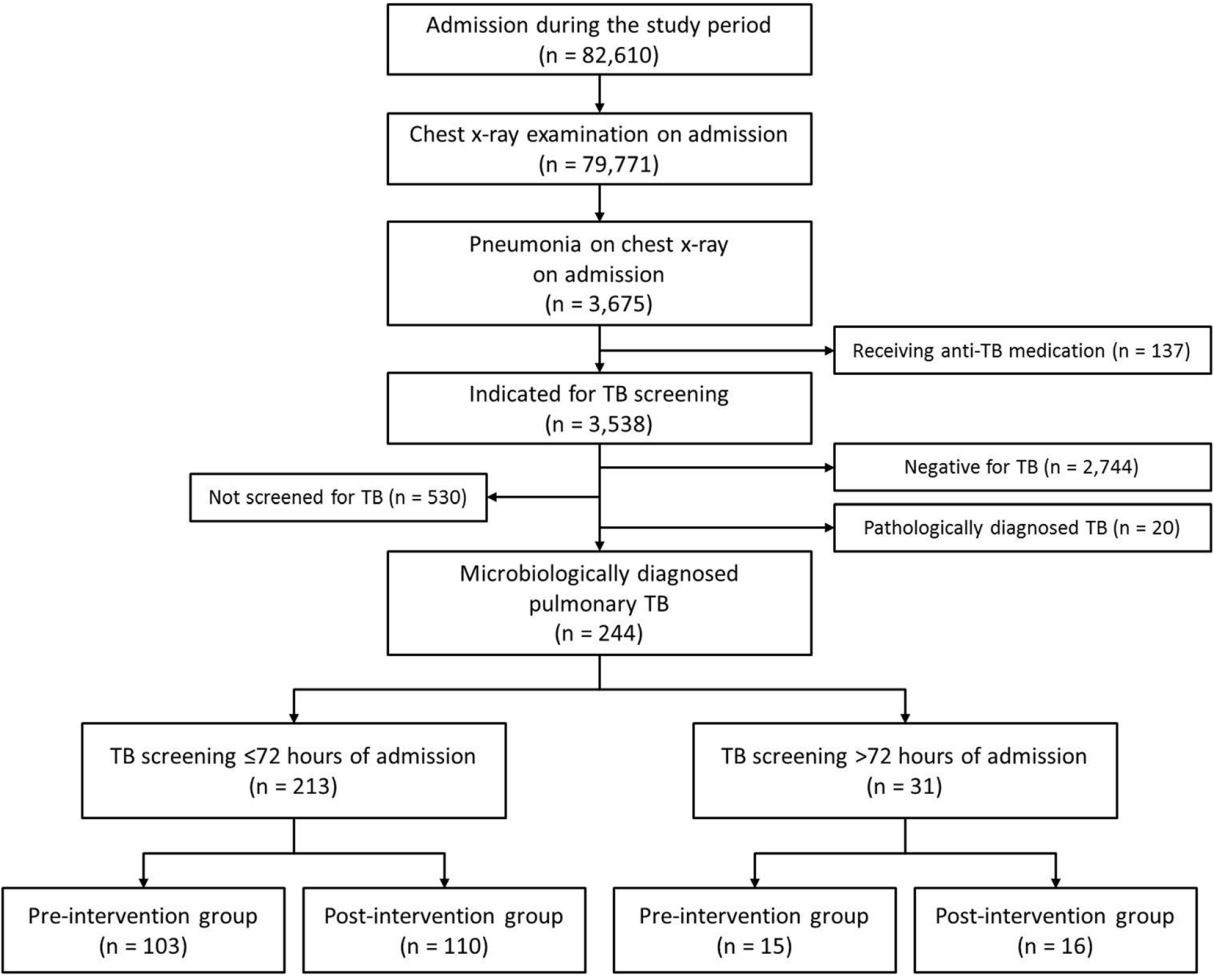
Flowchart for the inclusion of the study subjects.

During the study period, a total of 82,610 adult patients were admitted to the hospital, and 79,771 (96.6%) of them were examined with a CXR (Fig. 2). Among them, a total of 3,675 (4.6%) patients with pneumonia on CXR on admission were identified, and 137 (3.7%) of them had been receiving anti-TB medication for recently diagnosed TB (Fig. 2). Among the 3,538 patients indicated for pulmonary TB screening, 244 patients (6.9%) were microbiologically diagnosed with pulmonary TB, excluding 530 (15.0%) patients who were not screened during their hospitalization, 2744 (77.4%) patients who were negative for pulmonary TB, and 20 (0.6%) patients who were pathologically diagnosed without microbiological evidence for TB. Microbiological diagnosis of TB was made based on MTB culture plus PCR or Xpert (n = 174), MTB culture (n = 52), either PCR (n = 7) or Xpert (n = 5) or both (n = 6). Of these 244 patients diagnosed with pulmonary TB, 213 (87.3%) patients were timely screened (≤ 72 h of admission). Timely screened patients were more likely to be admitted to the departments of pulmonary or infectious diseases or thoracic surgery than other departments (Odds ratio [OR] 3.6; 95% confidence interval [CI] 1.5, 8.7; *p* = 0.005) and to have symptoms consistent with pulmonary TB (OR 3.2; 95% CI 1.3, 8.0; *p* = 0.011), and less likely to be immune compromised (OR 0.4; 95% CI 0.1, 0.9; *p* = 0.037) compared to those screened > 72 h of admission. The proportions of timely screened patients increased during the post-intervention period as compared to in the pre-intervention period (78.2% [1520/1943] vs. 73.0% [1164/1595]; *p* < 0.001) among those indicated for pulmonary TB screening. However, the proportions of timely screened patients in patients diagnosed with pulmonary TB were similar between the two study periods (87.3% [110/126] vs. 87.3% [103/118]; *p* = 0.997). Drug-resistant TB was identified in six (2.5%) patients: extensively drug-resistant TB in one and isoniazid-resistant TB in five patients.

### Comparison of characteristics between the pre- and post-intervention periods

The characteristics of all patients diagnosed with pulmonary TB and timely screened patients were summarized in Table 1. Their median age was 71 years (range 19–97), and 92 (37.7%) patients were ≥ 80 years old. There were no significant differences in distributions of sex and age, accompanying symptoms, and TB-suspected radiological abnormalities between the pre- and post-intervention periods (Table 1). The median of time-to-screen was 12.3 h (interquartile range [IQR]: 4.2–31.4), and this did not significantly differ between the pre- and post-intervention periods. The median of turnaround time (TAT) of the Xpert was 4.8 h (IQR: 3.4–8.4), which was significantly shorter than that of the PCR (*p* < 0.001) and smear microscopy (*p* < 0001; Table 2). Time-to-isolation was significantly reduced during the post-intervention period (median: 22.6 h; IQR: 2.3–75.7) compared to that during the pre-intervention period (median: 67.7 h; IQR: 20.9–219.8; *p* < 0.001). Consistent with this finding, patients were more likely to be isolated within 8 h after admission during the post-intervention period than those during the pre-intervention period (*p* < 0.001; Table 2). Among patients who were timely screened, time-to-isolation was further reduced during the post-intervention period (median of 15.1 h; IQR: 1.1–50.9, Table 2). Otherwise, the results were similar to those for all patients diagnosed with pulmonary TB.

**Table 1:** Comparison of demographics and clinical characteristics between the pre- and post-intervention periods.

Factor	All patients with pulmonary TB (n = 244)			Timely screened patients with pulmonary TB (n = 213)		
	Pre-intervention period (n = 118)	Post-intervention period (n = 126)	*P*	Pre-intervention period (n = 103)	Post-intervention period (n = 110)	*P*
Male sex	77 (65.3)	76 (60.3)	0.426	69 (67.0)	67 (60.9)	0.356
Age (years)	74 (59–82)	77 (65–82)	0.240	73 (58–81)	77 (64–82)	0.168
**Presenting symptoms and signs**						
Symptoms consistent with pulmonary TB	87 (73.7)	88 (69.8)	0.500	80 (77.7)	82 (74.6)	0.593
Cough of any duration	69 (58.5)	68 (54.0)	0.478	65 (63.1)	65 (59.1)	0.548
Cough lasting longer than 2 weeks	38 (32.2)	28 (22.2)	0.079	35 (34.0)	28 (25.5)	0.173
Weight loss	24 (20.3)	17 (13.5)	0.153	23 (22.3)	13 (11.8)	0.041
Night sweats	5 (4.2)	10 (7.9)	0.229	5 (4.9)	9 (8.2)	0.327
Fever ≥ 38 °C	30 (25.4)	31 (24.6)	0.882	28 (27.2)	30 (27.3)	0.988
TB-suspected radiological findings	77 (65.3)	79 (62.7)	0.378	69 (67.0)	71 (64.6)	0.707
Consolidation	54 (45.8)	45 (35.7)	0.110	49 (47.6)	42 (38.2)	0.166
Nodule	44 (37.3)	57 (45.2)	0.208	39 (37.9)	50 (45.5)	0.262
Effusion	33 (28.0)	20 (15.9)	0.022	29 (28.2)	19 (17.3)	0.057
Ground glass opacity	14 (11.9)	16 (12.7)	0.843	12 (11.7)	14 (12.7)	0.810
Cavity	23 (19.5)	27 (21.4)	0.708	21 (20.4)	23 (20.9)	0.925
Miliary nodules	3 (2.5)	7 (5.6)	0.336	1 (1.0)	7 (6.4)	0.066
Previous history of tuberculosis	33 (28.0)	29 (23.0)	0.306	29 (28.2)	24 (21.8)	0.285
**Comorbidity**						
Charlson comorbidity index ≥ 3	15/109 (13.8)	19/114 (16.7)	0.546	12/96 (12.5)	14/102 (13.7)	0.799
Diabetes mellitus	29 (24.6)	43 (34.1)	0.102	25 (24.3)	35 (31.8)	0.221
COPD	19 (16.1)	21 (16.7)	0.905	17 (16.5)	17 (15.5)	0.834
Cerebrovascular disease	18 (15.3)	28 (22.2)	0.164	15 (14.6)	23 (20.9)	0.227
Cardiovascular disease	15 (12.7)	16 (12.7)	0.997	12 (11.7)	13 (11.8)	0.970
Immune suppressed states*	21 (17.8)	21 (16.7)	0.815	16 (15.5)	15 (13.6)	0.695
HIV infection	1 (0.9)	0 (0.0)	0.300	1 (1.0)	0 (0.0)	0.300

Data are numbers (%) or medians (interquartile range).

*TB* tuberculosis, *COPD* chronic obstructive pulmonary disease, *HIV* human immunodeficiency virus.

*Immune suppressed states included connective tissue disease, leukemia, lymphoma, and solid tumor.

**Table 2:** Comparison of tuberculosis screening test results and clinical outcomes between the pre- and post-intervention periods.

Factor	All patients with pulmonary TB (n = 244)			Timely screened patients with pulmonary TB (n = 213)		
	Pre-intervention period (n = 118)	Post-intervention period (n = 126)	*P*	Pre-intervention period (n = 103)	Post-intervention period (n = 110)	*P*
**Respiratory specimen for screening**						
Sputum	109 (92.4)	116 (92.1)	0.928	95 (92.2)	100 (90.9)	0.728
Bronchial washing fluid	9 (7.6)	10 (7.9)	0.928	8 (7.8)	10 (9.1)	0.728
**Positive screening results**						
Smear microscopy	63 (53.4)	46 (36.5)	0.008	47 (45.6)	37 (33.6)	0.073
PCR*	81 (69.8)	81 (68.1)	0.771	61 (62.2)	64 (67.4)	0.456
Xpert MTB/RIF**	NA	70 (70.7)	NA	NA	62 (70.5)	NA
Time-to-screen (hours)	13.2 (4.5–36.6)	12.3 (3.9–27.4)	0.341	10.0 (4.2–23.2)	7.7 (3.7–18.7)	0.242
**Turnaround time (hours)**						
Smear microscopy	47.5 (15.6–74.9)	46.1 (18.5–68.2)	0.750	49.1 (15.6–89.5)	48.8 (23.5–71.8)	0.672
PCR	43.8 (31.2–64.1)	41.7 (24.7–60.0)	0.261	42.7 (32.2–64.0)	42.8 (27.0–61.7)	0.950
Xpert MTB/RIF	NA	4.8 (3.4–8.4)	NA	NA	5.3 (3.4–8.9)	NA
**Isolation**						
No isolation during the hospitalization	26 (22.0)	17 (13.5)	0.080	22 (21.4)	12 (10.9)	0.037
Pre-emptive isolation	18 (15.3)	28 (22.2)	0.164	18 (17.5)	28 (25.5)	0.157
**Time-to-isolation**						
Hours	69.7 (20.9–219.8)	22.6 (2.3–75.7)	< 0.001	53.7 (10.8–148.6)	15.1 (1.1–50.9)	< 0.001
< 8 h	24 (20.3)	52 (41.3)	< 0.001	24 (23.3)	52 (47.3)	< 0.001
< 72 h	36 (30.5)	41 (32.5)	< 0.001	36 (35.0)	38 (34.6)	< 0.001
≥ 72 h	58 (49.2)	33 (26.2)	< 0.001	43 (41.8)	20 (18.2)	< 0.001
**Anti-TB treatment**						
Anti-TB medication	104 (88.1)	122 (96.8)	0.009	91 (88.4)	107 (97.3)	0.011
Time-to-treatment (days)	4.5 (1.4–15.9)	2.3 (0.4–6.4)	0.003	3.5 (1.3–12.5)	1.6 (0.4–4.3)	< 0.001
Death attributable to TB	13 (11.0)	12 (9.5)	0.701	12 (11.7)	10 (9.1)	0.540

*TB* tuberculosis, *PCR* polymerase chain reaction, *NA* not applicable.

*During the pre- and post-intervention periods, the PCR test was performed among 116 and 119 of all patients with pulmonary TB and among 98 and 95 of timely screened patients.

**The Xpert was performed in 99 of all patients with pulmonary TB and in 88 of timely screened patients.

In terms of clinical outcomes, 226 (92.6%) patients received anti-TB treatment, and 25 (10.3%) patients, including seven (3.3%) patients who died before the diagnosis, died of pulmonary TB during their hospitalization. Patients in the post-intervention period received anti-TB treatment more frequently and earlier than those in the pre-intervention period although the mortality attributable to TB was not significantly different between the two study periods (Table 2). In a multivariate analysis, ≥ 80 years of age (OR 3.7; 95% CI 1.5–9.0; *p* = 0.005) and immune suppressed states (OR 3.5; 95% CI 1.4–9.0; *p* = 0.009) were significantly associated with in-hospital mortality attributable to TB.

### Impact of the Xpert MTB/RIF assay on time-to-isolation

**Figure 3:**
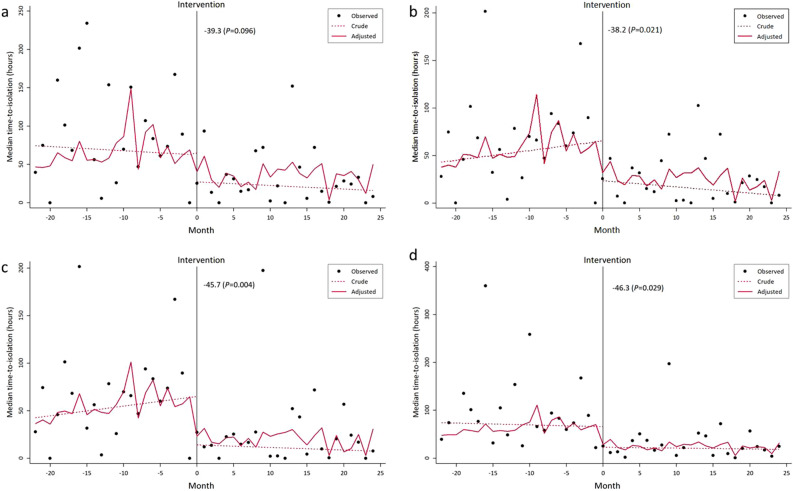
Trend of changes in time-to-isolation during the study period before and after introducing the Xpert MTB/RIF assay for screening TB in all patients diagnosed with pulmonary TB (**a**) and other patient subgroups: Group I (**b**), Group II (**c**), and Group III (**d**). Patient Group I included all of the timely screened patient; Patient Group II included all of the timely screened patients except for those not screened with the Xpert in the post-intervention period; Patient Group III included all of the timely screened patients except for those pre-emptively isolated during the whole study period and those not screened with the Xpert in the post-intervention period.

Time-to-isolation was significantly reduced during the post-intervention period (− 47.0 h; 95% CI − 74.1, − 19.9; *p* = 0.001) in a univariate quantile regression analysis. Other variables associated with time-to-isolation were age (0.80 h/year; 95% CI 0.01, 1.6; *p* = 0.049), the presence of TB-consistent symptoms (− 47.3 h; 95% CI − 86.2, − 8.4; *p* = 0.017), TB-suspected radiological findings (− 44.5 h; 95% CI − 76.1, − 12.9; *p* = 0.006), and higher Charlson comorbidity index (17.4 h per score; 95% CI 9.1–25.7; *p* < 0.001). For all patients with pulmonary TB, time-to-isolation was considerably reduced at the time of introducing the Xpert (− 39.3 h; 95% CI − 85.6, 7.0; *p* = 0.096) and maintained afterwards in multivariate segmented regression analysis although the statistical significance was not reached (Table 3; Fig. 3a). For patients who were timely screened (Group I), the time trend of decreasing time-to-isolation was significant. During the pre-intervention period, a trend in time-to-isolation nearly plateaued (1.0 h/month; 95% CI − 1.7, 3.7; *p* = 0.462), whereas a significant decrease in time-to-isolation (− 41.9 h; 95% CI − 82.2, − 1.7; *p* = 0.041) at the intervention point was observed, and a decreasing trend (− 0.6 h/month; 95% CI − 1.7, 0.5; *p* = 0.246) was maintained during the post-intervention period in the unadjusted segmented regression analysis. In a multivariate segmented regression analysis, a significant step change (− 38.2 h; 95% CI − 70.6, − 5.8; *p* = 0.021) and a decreasing trend during the post-intervention period (− 0.4 h/month; 95% CI − 1.3, 0.5; *p* = 0.368) were similarly observed (Table 3; Fig. 3b). In the Group II (n = 191) and Group III (n = 150), the results were similar with the trend of time-to-isolation being more reduced at the intervention point (Table 3; Fig. 3c,d).

**Table 3:** Trends in time-to-isolation before and after introducing the Xpert MTB/RIF assay for screening TB using a segmented quantile regression analysis, adjusted for demographics and factors associated with time-to-isolation.

Variable	All patients with pulmonary TB		Patient Group I		Patient Group II		Patient Group III	
	Coefficient (95% CI)	*P*	Coefficient (95% CI)	*P*	Coefficient (95% CI)	*P*	Coefficient (95% CI)	*P*
Pre-intervention trend (per month)	0.7 (− 2.8, 4.3)	0.683	1.0 (− 1.2, 3.3)	0.376	1.1 (− 1.2, 3.2)	0.342	0.9 (− 2.7, 4.4)	0.635
Post-intervention step change	− 39.3 (− 85.6, 7.0)	0.096	− 38.2 (− 70.6, − 5.8)	0.021	− 45.7 (− 76.4, − 15.0)	0.004	− 46.3 (− 87.6, − 4.9)	0.029
Post-intervention trend (per month)	− 0.1 (− 1.4, 1.1)	0.867	− 0.4 (− 1.3, 0.5)	0.368	− 0.1 (− 0.8, 0.6)	0.748	− 0.2 (− 1.5, 1.1)	0.756
Age	0.1 (− 0.3, 0.5)	0.779	0.1 (− 0.2, 0.3)	0.706	0.1 (− 0.3, 0.4)	0.737	0.0 (− 0.4, 0.5)	0.872
Charlson comorbidity index	11.3 (− 4.1, 26.6)	0.151	7.4 (0.7, 14.2)	0.031	5.8 (0.2, 11.5)	0.044	5.6 (− 3.9, 15.2)	0.245
Pulmonary TB symptoms	− 28.7 (− 74.9, 17.6)	0.223	− 11.6 (− 37.5, 14.2)	0.376	− 6.1 (− 30.7, 18.5)	0.626	− 14.9 (− 52.2, 22.5)	0.433
TB-suspected radiological findings	− 26.2 (− 58.8, 6.4)	0.115	− 25.8 (− 44.9, − 6.6)	0.009	− 24.5 (− 42.5, − 6.5)	0.008	− 18.1 (− 20.4, 149.4)	0.081

Patient Group I included all of the timely screened patient; Patient Group II included all of the timely screened patients except for those not screened with the Xpert in the post-intervention period; Patient Group III included all of the timely screened patients except for those pre-emptively isolated during the whole study period and those not screened with the Xpert in the post-intervention period.

*TB* tuberculosis, *CI* confidence interval.

### Sensitivity and specificity of screening tests for diagnosing pulmonary tuberculosis

For the 3008 patients who were screened for pulmonary TB, the Xpert, PCR, and smear microscopy were performed in 1125, 2549, and 2994 patients, respectively. For identifying MTB culture-positive pulmonary TB, the sensitivities of the Xpert (76.6%; 95% CI 64.3, 86.2) and PCR (73.4%; 95% CI 66.7, 79.4) were significantly higher than that of smear microscopy (47.8%; 95% CI 41.1, 54.5; *p* < 0.001 and *p* < 0.001, respectively; Table 4). In smear-positive and smear-negative patients, the sensitivity of the Xpert was 96.4% (95% CI 81.7, 99.9) and 61.1% (95% CI 43.5, 76.9), respectively. The specificities of the Xpert, PCR, and smear microscopy were similarly high.

**Table 4:** Diagnostic performance of the Xpert MTB/RIF assay, polymerase chain reaction test, and smear microscopy for culture-proven pulmonary tuberculosis.

Subject	Xpert			PCR	Smear microscopy
	Overall	Smear (+)	Smear (−)		
**Timely screened patients**					
Sensitivity (%) 95% CI	76.3 (45/59) 63.1–86.0	96.2 (25/26) 80.4–99.9	60.6 (20/33) 42.1–77.1	71.2 (116/163) 63.6–78.0	44.7 (80/179) 37.3–52.3
Specificity (%) 95% CI	98.2 (961/979) 97.1–98.9	81.8 (18/22) 59.7–94.8	98.5 (943/957) 97.6–99.2	98.8 (2,127/2,153) 98.2–99.2	98.5 (2,383/2,419) 97.9–99.0
PPV (%) 95% CI	71.4 (45/63) 58.5–81.8	86.2 (25/29) 68.3–96.1	58.8 (20/34) 40.7–75.4	81.7 (116/142) 74.3–87.7	69.0 (80/116) 59.7–77.2
NPV (%) 95% CI	98.6 (961/975) 97.5–99.2	94.7 (18/19) 74.0–99.9	98.6 (943/956) 97.7–99.3	97.8 (2,127/2,174) 97.1–98.4	96.0 (2,383/2,482) 95.2–96.7
**All patients with pulmonary TB**					
Sensitivity (%) 95% CI	76.6 (49/64) 64.3–86.2	96.4 (27/28) 81.7–99.9	61.1 (22/36) 43.5–76.9	73.4 (146/199) 66.7–79.4	47.8 (108/226) 41.1–54.5
Specificity (%) 95% CI	98.0 (1,040/1,061) 97.0–98.8	81.8 (18/22) 59.7–94.8	98.3 (995/1,012) 97.3–99.0	99.3 (2,334/2,350) 98.9–99.6	100.0 (2,717/2,718) 99.8–100.0
PPV (%) 95% CI	70.0 (49/70) 57.9–80.4	87.1 (27/31) 70.2–96.4	56.4 (22/39) 39.6–72.2	90.1 (146/162) 84.5–94.2	99.1 (108/109) 95.0–100.0
NPV (%) 95% CI	98.6 (1,040/1,055) 97.7–99.2	94.7 (18/19) 74.0–99.9	98.6 (995/1,009) 97.7–99.2	97.8 (2,334/2,387) 97.1–98.3	95.8 (2,717/2,835) 95.0–96.5

PCR, polymerase chain reaction; CI, confidence interval; PPV, positive predictive value; NPV, negative predictive value.

## Discussion

This study demonstrated that the application of the Xpert as a screening test significantly reduced time-to-isolation in patients with pulmonary TB. A higher number of patients with pulmonary TB were detected and isolated earlier after introducing the Xpert than before because the Xpert has shorter TAT and higher sensitivity than smear microscopy. After accounting for the time trend during the study period, the impact of the Xpert in reducing time-to-isolation was still significant.

The Xpert showed higher sensitivity and comparable specificity for TB diagnosis with smear microscopy in previous studies^5,6,10,11^. Furthermore, the Xpert can be performed in a short hands-on time, and requires minimal training, which might allow point-of-care positioning even in settings with limited resources^7^. In a developed country with a low TB incidence, the Xpert was cost-effective for reducing unnecessary isolation of TB-suspected patients^12^. Its use also reduced the duration of isolation and hospitalization and medical costs of TB-suspected patients^9,13–15^. However, in countries with a high or intermediate TB incidence, early isolation of patients with TB rather than early de-isolation of TB-suspected patients should be more emphasized for the prevention of in-hospital TB transmission. In South Korea where the TB burden is intermediate, a continuous influx of patients with pulmonary TB into hospitals can increase the risk of in-hospital TB exposure among hospitalized patients and HCWs. Moreover, the TB incidence was highest among older patients who often present with atypical symptoms and radiological findings, which could make early diagnosis of TB and early isolation more challenging^1,2,16^. In this study, the majority of patients were not pre-emptively isolated on admission and screening for pulmonary TB was more likely to be delayed in patients without symptoms consistent with pulmonary TB, suggesting the limitation of prediction of pulmonary TB based on clinical and radiological characteristics. Further, the median age of patients was 71 years, 28.3% of them had no TB-related symptoms, and 36.1% showed no TB-suspected radiological findings. This is particularly concerning as patients are often admitted in multi-bed rooms in South Korea, which might further facilitate the spread of TB in hospitals. Given that the Xpert provides more sensitive results with minimal technical expertise, the Xpert can be a more useful screening test for TB than smear microscopy. In addition, the strategy to screen patients with pneumonia for pulmonary TB can help timely identification of patients with pulmonary TB who are not clinically or radiologically suspected to have pulmonary TB during their initial presentation.

According to the Korea Disease Control and Prevention Agency guideline, individuals who stayed with a patient with active pulmonary TB in a closed space for more than 8 consecutive hours are potentially at risk of contracting TB, and initiation of a contact investigation for these individuals is recommended^17^. Although time-to-isolation was markedly reduced and the proportion of timely screened patients increased in the post-intervention period, less than half of the patients in the post-intervention period were isolated within 8 h of admission, and there were still a number of patients who were neither timely screened nor tested with the Xpert. A recent study showed that the introduction of the Xpert alone did not significantly increase the proportion of early isolated patients after admission in those with TB^16^. Therefore, multifaceted efforts, including continuous education and campaigns to improve compliance to the TB prevention strategy and to promote pre-emptive isolation of patients with suspected pulmonary TB should be performed in parallel with the application of the Xpert.

In this study, the Xpert demonstrated a sensitivity of 76.6%. Previous clinical field studies in South Korea reported the sensitivity of the Xpert (74.1% and 79.5%)^18,19^, which was lower than that (89%) in a meta-analysis including well-controlled clinical trials^5^. In our study, less than half of the included patients were positive in smear microscopy, suggesting that more than 50% of the study patients had low MTB burden in their respiratory specimens^4,18^. Considering that the semi-quantitative results of the Xpert correlated with the MTB burden in the respiratory specimen^11,19,20^, the low MTB burden of the these patients might reduce the sensitivity of the Xpert. The relatively low sensitivity of the Xpert in this study was concerning because the infectivity of TB patients with a negative Xpert result is not known. Thus, it is recommended that de-isolation of patients with negative Xpert results should be decided in conjunction with patients’ clinical and radiological findings.

Consistent with the previous study^6^, this study did not show a significant decrease of mortality despite early diagnosis and treatment of TB with the introduction of the Xpert. Old age was a significant factor for mortality attributable to TB in this study. This emphasizes that more efforts are needed for screening and early detecting patients with pulmonary TB among the elderly population on the primary care and community level in South Korea to prevent the elderly from visiting a referral hospital with progressed pulmonary TB. Screening for TB or latent TB infection in the elderly admitting to long term care facilities may be useful, and screening for latent TB infection seems to be more suitable in countries with a low/intermediate incidence of TB^21,22^. In South Korea, an annual TB screening for the elderly who are aged ≥ 65 years and the homeless population were established in 2018^23^. Its cost-effectiveness for reducing TB incidence among the elderly in the national, regional, and hospital levels should be determined in the future.

This study has some limitations. First, this study was conducted over a 4-year period, and thus, the HCWs’ increased awareness through continuing education and campaigns on early isolation of patients with TB might have contributed to the overall reduction in time-to-isolation. The proportion of pre-emptively isolated patients increased and time-to-screen was shortened during the post-intervention period. However, trends and step change were analyzed using a segmented regression analysis to account for such changes over time, which demonstrated a significant reduction in time-to-isolation after the introduction of the Xpert and a decreasing trend thereafter. Second, an economic analysis of the Xpert for deciding isolation based on its positive result was not examined. The cost-effectiveness of the Xpert should be performed for a future study in perspectives of reduction of in-hospital TB transmission and consequent investigation of TB contacts, and clinical outcomes of patients with TB. Third, this was a single-center study, making generalization of results to other healthcare settings challenging. However, this study may provide a strategy to limit in-hospital TB transmission, which can be potentially applicable in countries with an intermediate TB incidence.

In conclusion, the introduction of the Xpert as a screening test for pulmonary TB promoted the early diagnosis and isolation of patients with pulmonary TB. Application of the Xpert for TB screening is expected to reduce in-hospital TB transmission as well as the cost and labor for investigation of TB contacts.

## Materials and methods

### Study design and patients

Daejeon St. Mary’s Hospital is a 630-bed, university-affiliated hospital having four AIIRs in the hospital wards and two AIIRs in the emergency department, in Daejeon, South Korea. Daejeon has a population of 1.5 million people and this hospital has about 24,300 admissions per year. A multifaceted strategy for early identification and prompt isolation of hospitalized patients with pulmonary TB was implemented in January 2015 (Fig. 1): (1) prompt pre-emptive isolation and diagnostic tests in patients with clinically suspected pulmonary TB; (2) screening for pulmonary TB among hospitalized patients with pneumonia regardless of clinical or radiological suspicion; (3) regular education and campaigns to increase the compliance of HCWs to this strategy; and (4) direct notification of positive screening results to the attending physicians using a text message since April 2016. CXR was routinely performed in hospitalized patients before or at the time of admission, except for those who planned for a minor surgery/procedure or short-term hospitalization. In November 2016, the Xpert was introduced and has been used as a TB screening test since then.

We conducted a retrospective cohort study of adult inpatients (aged ≥ 18 years) screened for pulmonary TB from January 2015 to December 2018. Among this cohort, patients who were microbiologically diagnosed with pulmonary TB (positive results for at least one of the Xpert, PCR, or MTB culture) were included for study analysis, and among them, those who were timely screened (≤ 72 h of admission) were also separately analyzed to exclude the effect of non-compliance to the strategy. Patients who were under anti-TB treatment for previously diagnosed TB and who were pathologically diagnosed without microbiological evidence of TB were excluded. Demographics, clinical characteristics, radiological study results (CXR and chest computed tomography), time-to-screen (time between a patient’s arrival and submission of the first specimen to the laboratory), time-to-isolation (time between a patient’s arrival and isolation of the patient in an AIIR or in a single room), TAT of each screening test (time between submission of the first specimen to the laboratory and reporting the test results), and time-to-treatment (time between a patient’s arrival and the initiation of anti-TB medication) were retrieved from electronic medical records. TB-suspected radiological findings were identified and classified based on radiologists’ reports. The study period was divided into two by the introduction of the Xpert: a pre-intervention period (January 2015 to October 2016) and a post-intervention period (November 2016 to December 2018). The primary outcome was time-to-isolation before and after the intervention (introduction of the Xpert for TB screening), and secondary outcomes were clinical outcomes and the sensitivities and specificities of the Xpert and other screening tests.

This study was approved by the Institutional Review Board of Daejeon St. Mary’s Hospital that waived the need for informed consent (Approval No.: DC19RESI0030). This study was performed in accordance with the Declaration of Helsinki and relevant guidelines and regulations.

### Microbiological tests

For TB screening tests, three consecutive sputum specimens with an interval of 8–24 h and/or single bronchial washing fluid specimen were collected from patients. All screening tests (smear microscopy, PCR, and the Xpert) and MTB culture were performed using the first sputum specimens collected simultaneously or single bronchial washing fluid specimen during patients’ stay in the hospital. Smear microscopy and MTB culture were performed for two additional sputum specimens.

Smear microscopy was performed using concentrated specimen following liquefication and decontamination with N-acetyl-L-cysteine-sodium hydroxide. Auramine-rhodamine fluorescence staining was used for acid-fast bacilli stain, confirmed by Ziehl–Neelsen staining. PCR tests were performed using a commercially available kit (AdvanSure TB/NTM real-time PCR kit, LG Life Science, Seoul, South Korea) according to the manufacturer’s recommendations. The Xpert was performed according to the manufacturer’s recommendations. If the initial result was invalid, re-testing was performed using the same specimen when the residual volume of the specimen was adequate. If the collected specimen was inadequate, re-sampling was requested. Smear microscopy (Monday to Saturday) and the PCR test (on Mondays, Wednesdays, and Fridays) were performed during working hours. The Xpert was performed daily anytime during working and duty hours.

For the MTB culture, solid (3% Ogawa medium, Asan Pharmaceutical Co., Seoul, South Korea) and liquid (BACTEC MGIT 960 system, Becton Dickinson diagnostic instrument systems, Sparks, MD, USA) media were used. Cultures on liquid media were continuously and automatically monitored in the incubator for 6 weeks, and cultures on solid media were monitored weekly for 8 weeks.

### Statistical analysis

Comparisons were performed using a Pearson χ2 test for categorical variables and Wilcoxon signed rank test for continuous variables. Because time-to-isolation was right-skewed, a quantile regression analysis was used to examine the crude effect of the intervention and variables on reducing time-to-isolation. To adjust for time trend and statistically significant confounding variables in the univariate analyses (*p* < 0.1) in assessing differences in step-change and monthly trend of time-to-isolation between the pre- and post-intervention periods, we performed multivariate segmented regression analyses. A 95% CI for the level and trend was obtained using a bootstrap.

Since not all of the patients were screened nor used the Xpert during the post-intervention period and pre-emptive isolation (isolation < 2 h of admission) was predicted to be more frequent during the post-intervention period than the pre-intervention period, the same analyses for the following three sub-groups were performed: all of the timely screened patients (Group I), all of the timely screened patients except for those not screened with the Xpert in the post-intervention period (Group II), and all of the timely screened patients except for those pre-emptively isolated during the whole study period and those not screened with the Xpert in the post-intervention period (Group III). Factors associated with delayed screening and TB-attributable in-hospital mortality were analyzed with a logistic regression analysis.

The sensitivity, specificity, and positive and negative predictive values of three types of screening tests were determined with 95% CIs and compared to one another using the results of MTB culture from simultaneously sampled specimens as a standard reference. Stata version 13.0 software (Stata Corporation, College Station, TX, USA) was used for all data analyses.

## Data Availability

The data is available only upon a reasonable request to the corresponding author.
